# The Utility and Limitations of Artificial Intelligence-Powered Chatbots in Healthcare

**DOI:** 10.7759/cureus.73127

**Published:** 2024-11-06

**Authors:** Jafar Hayat, Mohammad Lari, Mohammad AlHerz, Ali Lari

**Affiliations:** 1 Department of Surgery, Jaber Al-Ahmad Al-Sabah Hospital, Kuwait City, KWT; 2 Department of Orthopedic Surgery, Al-Razi National Orthopedic Hospital, Kuwait City, KWT; 3 Department of Anatomy, Trinity College Dublin, Dublin, IRL

**Keywords:** artificial intelligence, chatbot, chatgpt, healthcare, language-models

## Abstract

At the intersection of artificial intelligence (AI) and healthcare, it is essential that clinicians grasp the ability of chatbots. AI-powered chatbots such as ChatGPT are being explored for their potential benefits by both individuals and institutions. The utility of ChatGPT (OpenAI) in various scenarios was explored through a series of recorded prompts and responses. In the clinical aspects, the chatbot facilitated tasks such as triage, patient consultation, diagnosis, and administrative responsibilities. Their capacity to translate and simplify intricate medical topics was also evaluated. For research purposes, the chatbots' abilities to suggest ideas, prepare protocols, assist in manuscript writing, guide statistical analyses, and recommend suitable journals were assessed. In the educational domain, chatbots were tested for simplifying complex subjects, reviewing procedural steps, generating clinical scenarios, and formulating multiple-choice questions. A comprehensive literature review was also conducted across Medline, Embase, and Web of Science. Chatbots, when optimally employed, can serve as invaluable resources in healthcare, spanning clinical, research, and educational domains. Their potential lies in enhancing efficiency, guiding decision-making, and facilitating patient care and education. However, their application requires a nuanced understanding and caution regarding their limitations.

## Introduction

At the intersection of artificial intelligence (AI) and healthcare, it is vital for providers to grasp the currently transformative ability of chatbots. These tools can generate context-specific solutions across a broad spectrum of scenarios, thereby enhancing daily workflow and efficiency [1]. This article offers a look into the versatile applications of chatbots across various clinical, academic, and administrative duties and presents a perspective on how these AI tools, with proper prompting, can evolve into invaluable resources in healthcare.

## Materials and methods

This methodology aimed to draw a comprehensive view of current knowledge and practices around AI chatbots in healthcare. The prompts and questions were posed to the free 3.5 version of the AI chatbot, ChatGPT (OpenAI). The first responses obtained from the chatbot were recorded and discussed below. The primary outcome measure of this study was to assess and discuss the role of ChatGPT in several clinical scenarios highlighted in the figures below in the results section, and the secondary outcome measure involved the discussion of the role of chatbots in various aspects of healthcare in general, stratified by several subheadings. Findings were discussed descriptively and categorized into appropriate subheadings. No comparator or control was used for this observational study, and interventions provided by ChatGPT were compared to present standards of care found in the available literature.

For the literature review, a comprehensive search in relevant databases including Medline, Embase, and Web of Science was performed using keywords that included; “Chatbots”, “Language models”, “Artificial intelligence”, and “ChatGPT”. These were coupled with terms that investigate their medical application; “Research”, “Clinical”, “Education”, “Triage”, “Examination”, “Administration”, “Translation”, “Documentation” and “Healthcare”. An initial investigation into the applications of chatbots was conducted to identify common themes and trends in the recent application of language models, such as the GPT series by OpenAI, BERT by Google, and Claude. Thus, the selection criteria involved articles that directly addressed the use of AI-powered chatbots in healthcare, whether in patient care, administrative efficiency via patient scheduling, streamlined data collection or interpretation, billing, resource allocation, quality improvement, or educational support for medical professionals.

## Results

**Figure 1:**
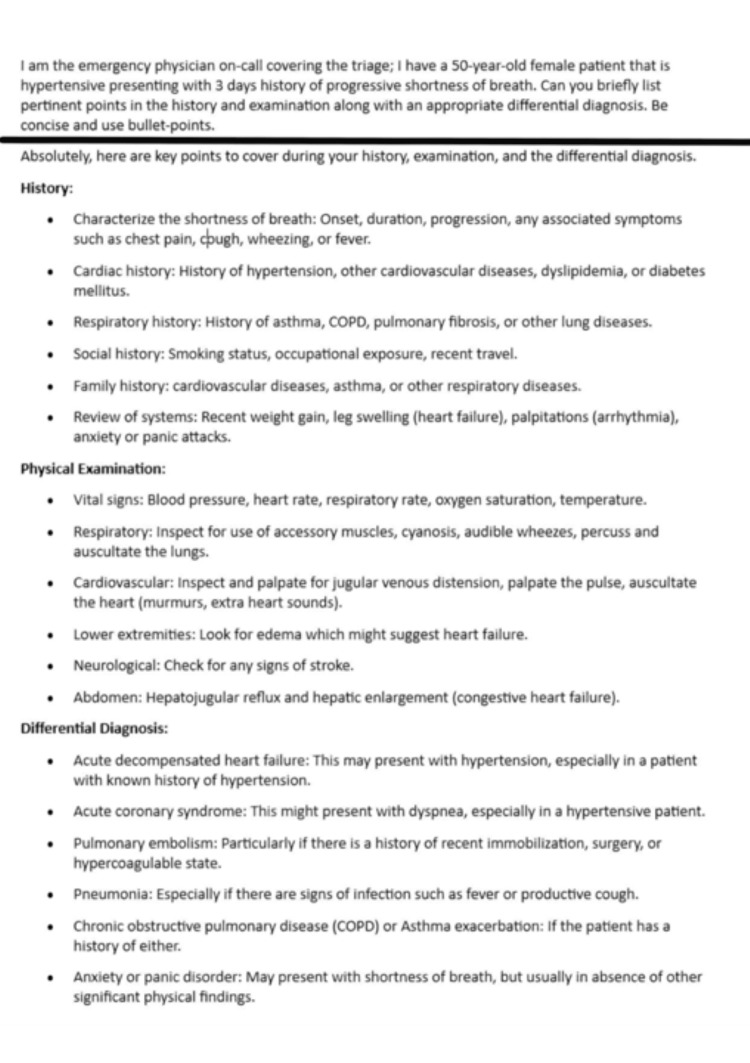
A prompt and a response from ChatGPT suggesting pertinent points in the history and examination.

AI-powered chatbots demonstrated significant utility in clinical practice and research. In clinical settings, chatbots assisted in triage and patient consultations by providing structured frameworks for history-taking (Figure 1) and suggesting differential diagnoses based on symptoms.

**Figure 2:**
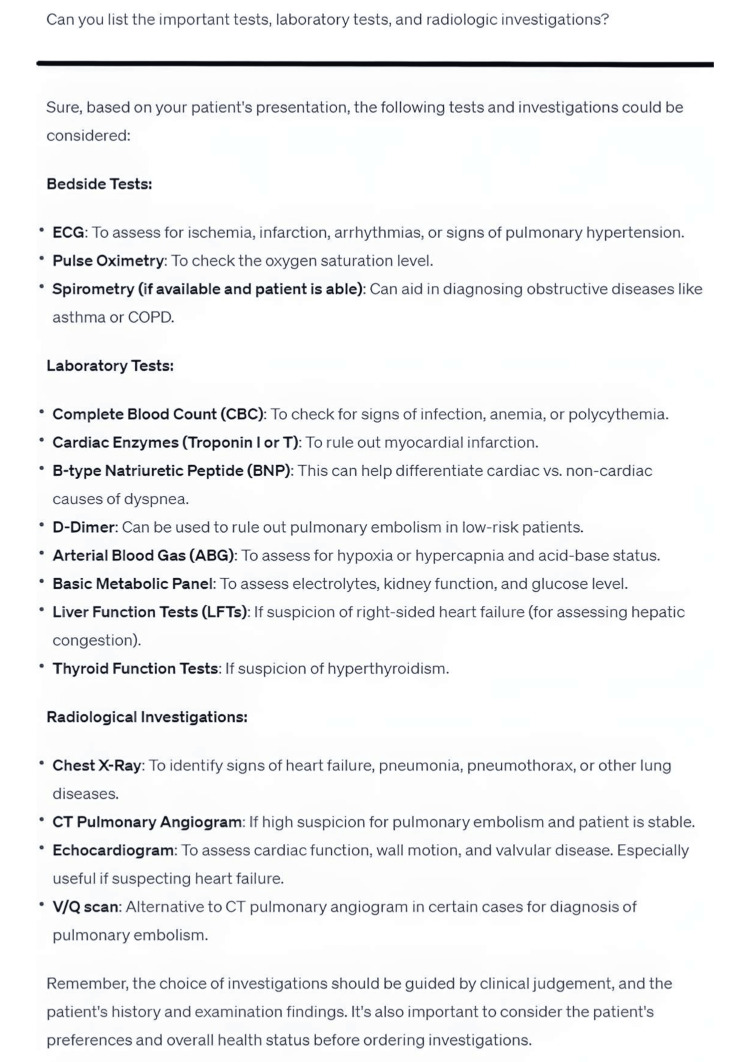
A follow-up prompt inquiring into the relevant investigations for the same patient.

**Figure 3:**
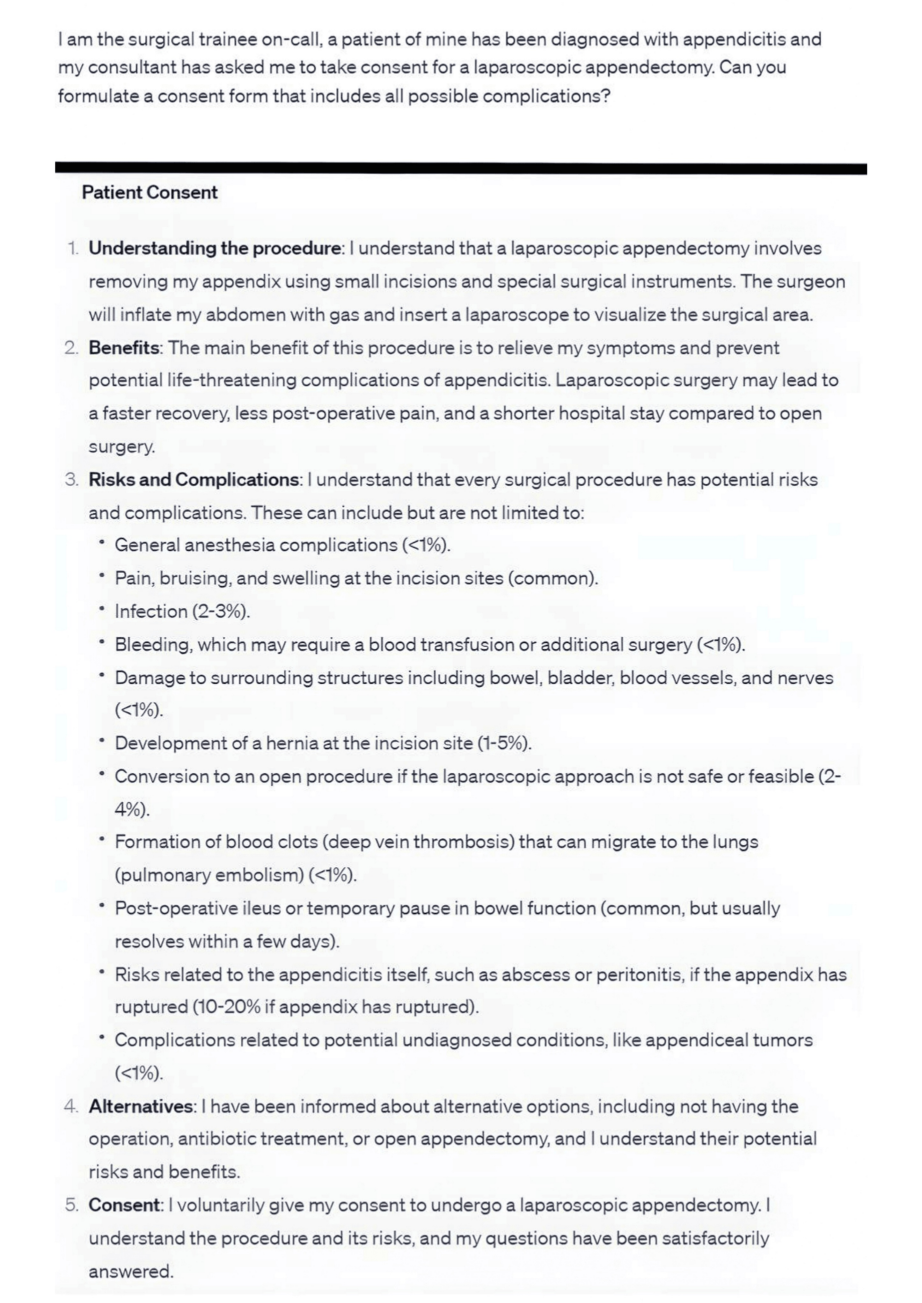
A prompt asking for the details regarding patient consent for a laparoscopic appendectomy procedure.

They recommended relevant investigations (Figure 2) and facilitated the patient consent process by generating clear, jargon-free forms, helping patients understand procedures, risks, and benefits more effectively (Figure 3).

**Figure 4:**
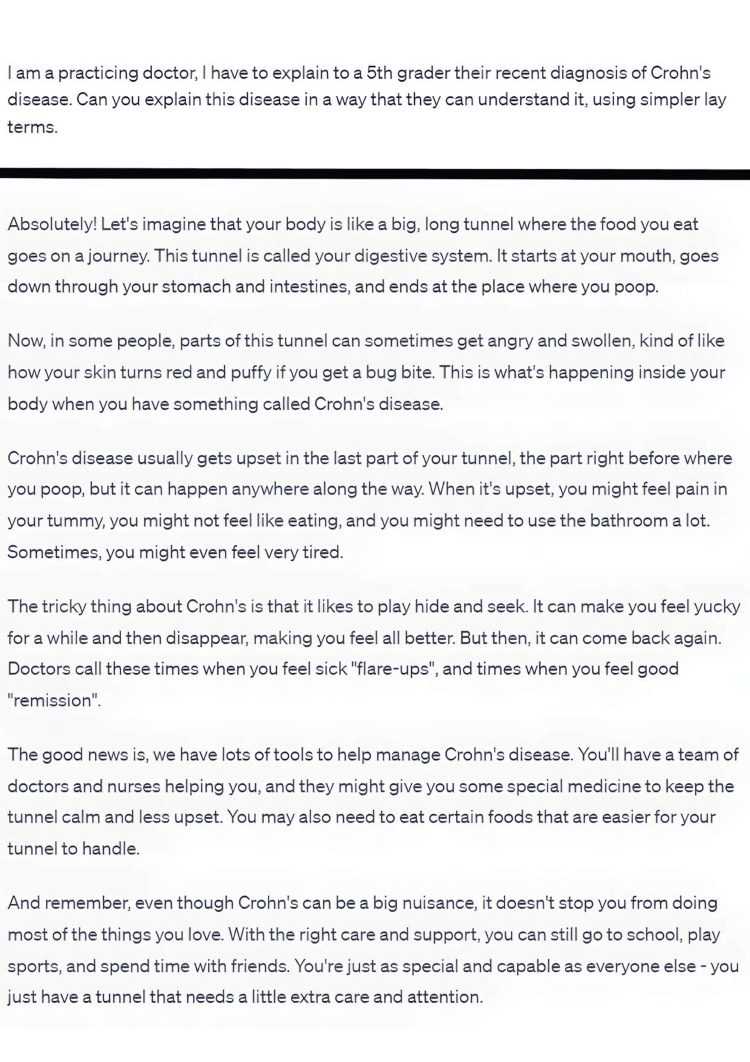
A prompt generating a response that uses metaphors and lay terms to easily explain Crohn’s disease to a child.

Chatbots further simplified medical language for diverse populations, aiding patient comprehension of diagnoses and treatments, especially for individuals of different ages and education levels (Figure 4). Additionally, chatbots reduced administrative burdens by aiding with documentation, dictation, and ensuring accurate record-keeping.

**Figure 5:**
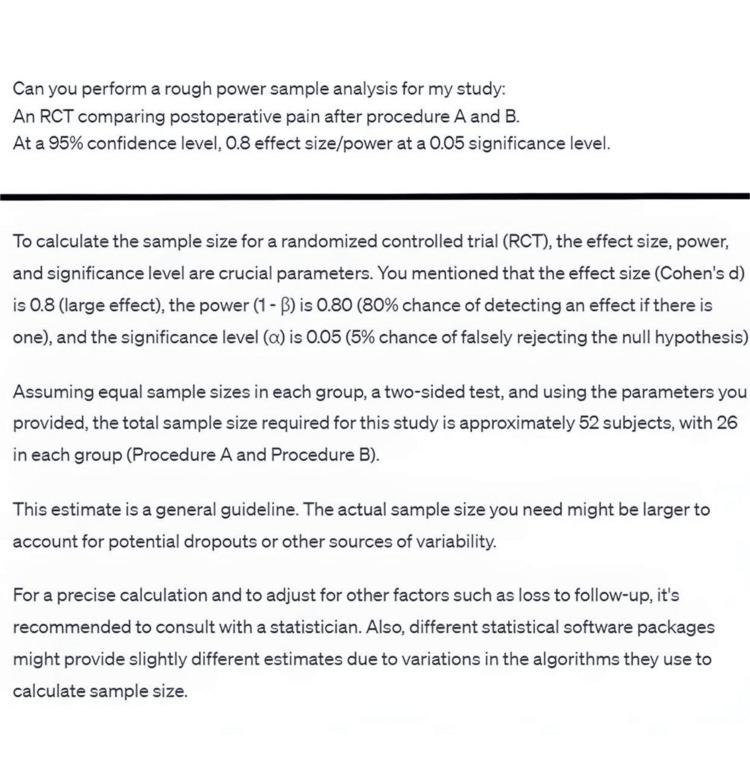
A brief power analysis performed by the chatbot. The chatbot often guides the user if information is missing.

**Figure 6:**
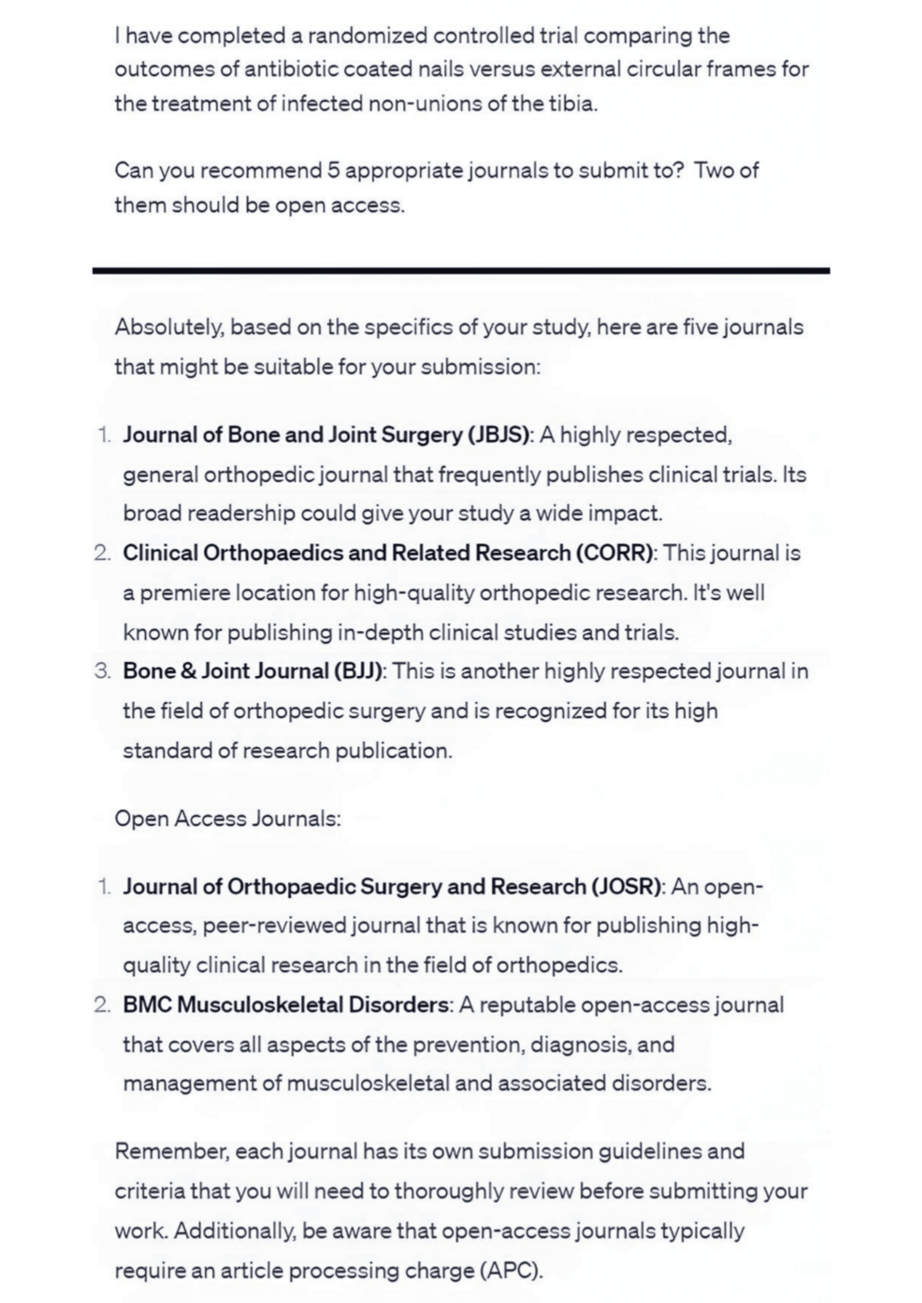
Suggestions for journals based on the brief information given to the chatbot.

In research, chatbots supported idea generation by identifying potential research areas and guiding clinicians in brainstorming systematic reviews on trending topics. They assisted in protocol preparation, generating comprehensive research protocols using predefined templates, and improved manuscript writing by refining structure and correcting language errors. Chatbots also guided researchers on statistical analysis and power sampling, though caution is necessary due to potential inaccuracies (Figure 5). Moreover, chatbots provided journal recommendations by matching the manuscript’s content with relevant journals (Figure 6).

**Figure 7:**
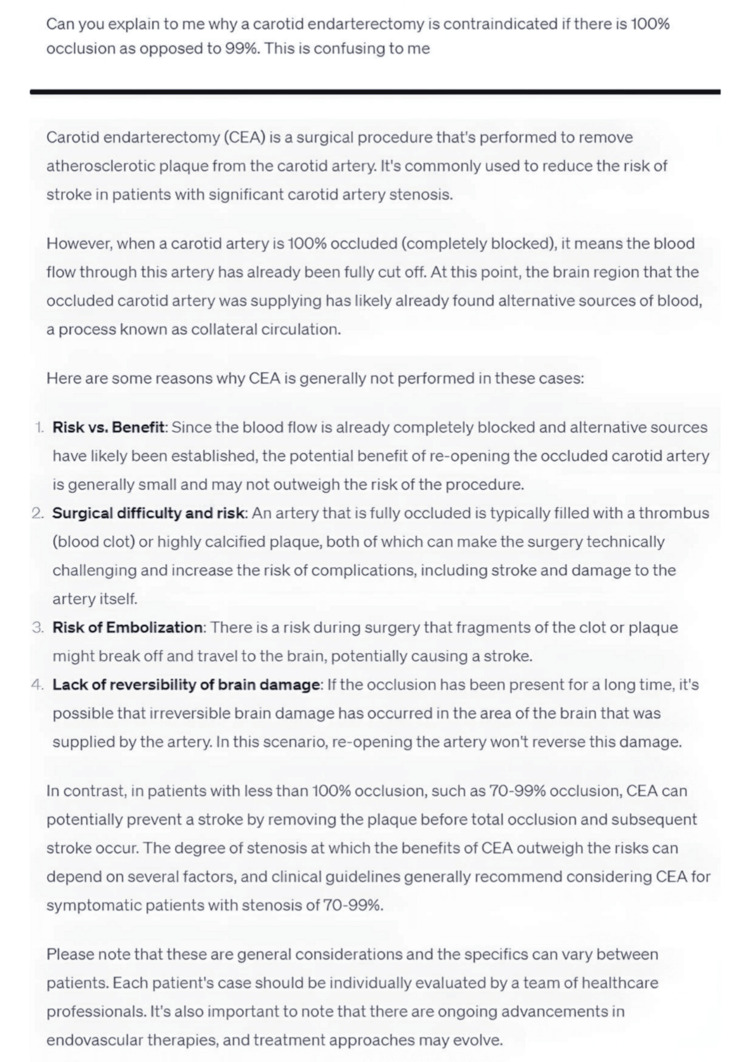
A question and answer displaying the response of the chatbot explaining the concept of why carotid endarterectomies are contraindicated beyond a certain degree of occlusion.

In education, chatbots efficiently summarized medications, including their mechanisms, doses, and potential interactions (Figure 7). They also reviewed procedures, offering detailed step-by-step explanations to help both beginners and experienced clinicians. Chatbots further generated clinical scenarios and multiple-choice questions (MCQs), enhancing interactive learning and self-assessment.

While chatbots offer valuable support, they can occasionally generate inaccurate information, necessitating human oversight for critical decisions.

## Discussion

What are chatbots?

Chatbots are programs that simulate human-like conversations. They utilize AI technology, natural language processing (NLP), and machine learning (ML) to assess the user input and provide an appropriate output (response) [1, 2]. When a chatbot is AI-powered, it is capable of handling complex conversations and can learn from different user interactions. This enables them to make sense of various types of unstructured input. This is in contrast to the earlier rule-based systems, which were only able to respond if a certain structured input was used. Voice-based chatbots represent AI-powered chatbots with the feature of voice-based interactions, allowing for a more natural conversation without the need for typing. 

How do they work?

While chatbots have certain limitations (limited diagnostic capabilities, a lack of empathy and human touch, an inability to conduct and appraise physical examinations, and technical limitations), users can achieve optimal responses by understanding the algorithmic flow of chatbot conversations. The user initiates the interaction by providing input, referred to as a prompt. This prompt serves as the main query and establishes the context for the chatbot's output. It is important to note that chatbot conversations are iterative, involving multiple interactions. This means that building context through multiple prompts can lead to the desired response. This has given attention to what has been termed “Prompt engineering,” which refers to refining instructions or prompts that effectively obtain various responses from chatbots [3]. Furthermore, chatbots can be fine-tuned to generate responses based on specific datasets and data domains, such as incorporating medical data into their database. By extracting responses from specialized data, chatbots ensure that their responses align with current medical information.

Clinical utility 

In the clinical setting, chatbots serve as invaluable tools, offering clinicians a plethora of advantages. These virtual assistants, akin to advanced search engines, seamlessly retrieve medical information, propose treatment algorithms, aid in patient counseling, and alleviate the burdens of administrative tasks. Particularly for junior doctors or clinicians in training, chatbots enable the creation of comprehensive tables to succinctly outline the pros and cons of various investigations, guiding the selection of the optimal next step in patient workups and presenting diverse treatment algorithms. This article, albeit briefly, will explore some of the rudimentary applications of chatbots, showcasing their transformative potential in healthcare.

Triage and Initial Patient Consultation, an On-call Assistant 

At an individual level, clinicians can utilize chatbots to analyze patient symptoms and facilitate the process of history taking. These chatbots intelligently examine the symptoms and offer clinicians a structured framework for conducting a thorough patient history and examination by using natural language processing, contextual analysis and symptom triaging, pattern recognition and matching with medical databases, and via the integration of clinical guidelines and best practices [4]. By referring to a list of potential differential diagnoses, the chatbot suggests pertinent questions to ensure important details are not overlooked [5, 6] (Figure 1). This interactive process allows clinicians to have ongoing conversations with the chatbot, incorporating new information and prompting the chatbot to provide more detailed responses. This collaborative approach combines the clinician's expertise with the analytical capabilities of the chatbot. However, there are instances where its application in emergency triage settings may be limited at this stage [7, 8].

Aiding Diagnoses and Providing General Work-up 

Healthcare professionals can employ chatbots to assist in the diagnostic process and subsequent patient work-up. Through dynamic interaction, clinicians can utilize chatbots' access to timely information to arrive at a tailored differential diagnosis. Additionally, clinicians can provide updated patient data, prompting the chatbot to suggest appropriate diagnoses and recommend relevant investigations [9]. For example, if a patient presents with respiratory symptoms, the chatbot can offer a list of pertinent questions and potential differential diagnoses [10]. Upon request, the chatbot can propose investigations to help narrow down the diagnosis and rule out more severe conditions (Figure 2). This dynamic process aids clinicians in structuring their approach and ensures comprehensive assessments during the initial patient consultation.

An Outline for Patient Consent 

Obtaining patient consent is a crucial aspect of a clinician's daily work, particularly for junior healthcare professionals. In this regard, chatbots can play a supportive role by assisting in the formulation of consent forms that utilize clear language and avoid medical jargon (Figure 3). These chatbots have the capability to search for relevant information related to a specific procedure, including its risks, benefits, and alternatives, and generate concise paragraphs that are easily understandable by patients [11]. This functionality offers two significant benefits. Firstly, it aids clinicians in effectively communicating the information using plain language, ensuring patients comprehend the important aspects of the procedure. Secondly, patients themselves can engage with the chatbot in an interactive dialogue, enabling them to actively participate in the consent process [11, 12]. Moreover, chatbots can easily create tailored consent forms for different procedures, saving time and effort for both clinicians and patients.

Administrative Burden

The day-to-day work of healthcare professionals can be demanding, particularly due to administrative tasks and clinical documentation [13]. Chatbots can act as virtual assistants, assisting in dictation and reducing the time and effort required for manual data entry [14]. They also offer the benefit of grammar and language error checking, ensuring accurate and well-structured documentation [15]. Chatbots have recently been assessed for their ability to write discharge summaries and operative notes, yielding encouraging results [16]. Additionally, chatbots can be prompted with voice commands to obtain information such as drug dosage or clarify confusing topics.

Explaining and Translating

Chatbots are capable to using simpler language to accommodate for different ages and levels of education [17]. This is useful when trying to explain diagnoses, treatment, medication and prognosis in simpler terms (Figure 4) [18]. Further, chatbots are efficient translators and are capable of translating different text to many different languages in a clear and concise manner [19]. 

Research utility 

The utility of AI-powered language models has garnered significant attention in recent times. It is clear that chatbots will have a significant role to play in future research, especially considering the rapid advancements in language models. However, there exist numerous legitimate concerns among researchers regarding the reliability and potential misuse of these language models in research [20, 21]. While there are various ways chatbots can assist in research on a larger scale, this article will primarily focus on discussing a selection of methods employed at the individual level. 

Suggesting Ideas

Chatbots can assist clinicians in identifying areas of medical interest. By engaging with chatbots, clinicians can brainstorm ideas and discover potential research areas that require further investigation, such as conducting systematic reviews on current trending topics [22]. However, it's worth noting that the availability of the latest online medical databases may limit the scope of this feature, potentially restricting it from serving as a source of inspiration.

Preparing Protocols

Chatbots can be valuable in aiding the preparation of research protocols. By utilizing predefined templates, clinicians can input their research ideas and methodology, allowing the chatbot to generate a comprehensive protocol. This functionality can be particularly helpful in ensuring completeness and saving time that would otherwise be spent on manual data entry [23].

Research Writing and Language

Chatbots can serve as helpful guides for structuring and formatting manuscripts. They can assist in editing the overall layout of the document while identifying and correcting linguistic and grammatical errors [20, 24]. Their efficiency in language processing enables them to provide prompt feedback on language-related issues. Chatbots can also be asked to formulate language in different writing styles. However, their ability to accurately write entire manuscripts while maintaining context appears to fall short [25].

Statistical Analysis and Power Sampling

Chatbots can provide guidance on various statistical analysis techniques, presenting explanations in simpler terms for better understanding. They can assist researchers in selecting appropriate analytic tests based on the research design and offer information on general statistical queries [26]. Additionally, chatbots have the capability to perform power analyses when provided with the necessary information, aiding researchers in determining the appropriate sample size for their studies (Figure 5). However, there have been reports of inaccuracies in generating power samples [27]. Thus, a person with statistical experience should be involved in overseeing analytical suggestions by the chatbot. 

Journal Recommendation

Chatbots can assist researchers in finding suitable journals for their manuscripts by analyzing the scopes of various journals and matching them with the title and premise of the article (Figure 6). By considering the content and focus of the research, chatbots can provide recommendations on journals that align well with the manuscript. Moreover, they can offer guidance on effective strategies for searching and identifying journals that are a good fit for the research topic and methodology. 

Studying and education utility 

Chatbots can aid in studying and reviewing information, particularly for medical students and clinicians in training. Often, users rely on standard search engines to gather information, but this can be overwhelming as they need to search through multiple sources to find concise summaries [28]. In contrast, chatbots have the ability to provide personalized responses that are brief and easily comprehensible, making the learning process more efficient and effective for users.

Simplifying complex topics can save time and effort while also potentially aiding in information retention [29]. Chatbots play a crucial role in providing quick summaries of various medications, including their doses, mechanisms of action, and safety profiles, even when considering potential interactions with other drugs [30]. This makes chatbots an excellent tool for extracting concise information rapidly (Figure 7).

Additionally, chatbots can assist in reviewing the steps involved in procedures and surgeries. They can provide detailed explanations of each step and the underlying reasons behind them. This functionality proves particularly useful for beginners, as it helps them understand basic concepts and review the procedure [29, 31]. Similarly, experienced clinicians can also benefit from chatbots by using them to review the steps involved in procedures they are already familiar with.

Chatbots possess the ability to formulate a wide range of clinical questions and patient scenarios, making them a valuable resource for both clinicians and students. By generating realistic scenarios across various topics, chatbots simulate real-world encounters and offer users an opportunity to apply their knowledge in practical settings [32]. These scenarios can be accompanied by pertinent questions encouraging users to think critically and provide accurate responses.

Moreover, chatbots can generate multiple-choice questions (MCQs) at varying levels of difficulty. This feature enables users to assess their understanding and reinforce key concepts through self-testing [33]. By offering a diverse range of clinical questions and patient scenarios, chatbots serve as an interactive learning tool that may enhance clinical reasoning and knowledge retention.

Limitations

While AI-powered chatbots offer convenience and impressive language comprehension, it is important to be aware of their limitations. Chatbots primarily function as advanced search engines, interpreting language at a complex level 1. However, they can occasionally misinterpret medical queries, lacking a complete understanding of the nuances that may result in inaccurate responses. This notable issue, termed as "hallucinations", involves these AI systems producing information that might not be based on the actual input data, potentially leading to misleading or false conclusions [34-36]. Clinicians should exercise caution when relying solely on chatbot-generated answers. Furthermore, it is important to note that chatbots do not have real-time access to the latest medical literature or complex online medical libraries. Therefore, their responses may not always reflect the most up-to-date information available. However, it is likely that this drawback will be addressed in the future. Another limitation of this narrative review is the narrow scope of replies this article covers, leaving room for variance in AI-generated results to other clinical scenarios. The risk of error should always be considered in developing technologies, and it is important to not rely on such technologies exclusively for both clinicians and patients alike.

To maintain a balanced approach, healthcare professionals should use chatbots as supplementary tools rather than relying solely on them. The expertise and clinical judgment of clinicians should not be overshadowed by the capabilities of chatbots. By recognizing these limitations, healthcare professionals can effectively utilize chatbots to enhance their practice without compromising patient care. Similarly, while the utility of chatbots in research is promising, addressing their limitations and concerns is essential. Ethical considerations arise regarding the use of chatbots in research and while there is no consensus on this matter currently, many journals require transparency in chatbot usage [37]. To mitigate the unregulated use of chatbots, chatbot detection programs exist, however their accuracy is uncertain. 

Moreover, chatbots rely on preexisting data up to a certain point in time, which means they cannot access the latest evidence without software extensions. Finally, researchers must independently verify the data extracted by chatbots and avoid routine unregulated use that may lead to inaccuracies.

Chatbot Extensions and Other Chatbots 

To address the current limitations of chatbots, various internet browser software offers features that enhance their capabilities. Specific software extensions can enable chatbots like ChatGPT to access real-time data and up-to-date information that reflects the latest developments in various fields. Additionally, there are extensions available that can generate concise summaries of articles or videos, condensing them into smaller paragraphs or bullet points. Furthermore, integrating chatbots with web browsers through an extension allows for the integration of the standard search engine with AI-powered language comprehension. These extensions may enhance the functionality and performance of chatbots. However, even in the absence of an extension, newer versions of ChatGPT are likely to add additional features to enhance their online outreach (GPT-4). 

The AI chatbot landscape in healthcare features several advanced platforms. ChatGPT (OpenAI Incorporated, Mission District, San Francisco, United States), which is discussed in this study, Google Bard (Alphabet Inc., CA, US), and Microsoft Bing (Microsoft Corporation, WA, US). Google's Bard is tailored to generating contextually rich responses, potentially improving patient engagement and understanding in healthcare interactions. Microsoft's Bing search capabilities offer a broad spectrum of current medical knowledge, which is useful for up-to-date clinical decision-making [38]. However, direct comparisons are scarce, and results from their performance in exams are similar [39, 40]. In patient-specific healthcare, Ada Health and Babylon Health are often cited [41]. Ada Health's strength lies in its ability to conduct detailed symptom analyses and guide initial patient assessments using a vast medical database [42, 43]. Babylon Health, on the other hand, specializes in virtual healthcare delivery, offering remote consultations and personalized health advice, aligning with the trend toward telemedicine [41]. Nevertheless, the majority of surfacing research has focused on ChatGPT, likely due to the ease of access.

## Conclusions

Exploring AI-powered language chatbots like ChatGPT underscores their potential in healthcare and research. These digital tools can assist in clinical work, reduce administrative burden, and enhance educational platforms. Nevertheless, the adoption of such technology necessitates a balanced view, acknowledging their current limitations, especially in context-specific situations requiring a creative approach to problem-solving. Looking forward, the evolution of chatbots could see them becoming more integral to personalized healthcare, with advancements potentially enabling these tools to offer more nuanced, patient-specific insight. Thus, knowledge and proficiency in chatbot usage are likely to offer an advantage to the daily work of the healthcare provider. The rapid pace and trajectory of AI language model development suggest that chatbots might soon evolve beyond their current role as supplementary tools in healthcare. Although there are significant barriers to the widespread implementation of AI technology in healthcare, chatbots have already proven to be useful to healthcare providers in various ways. Clearly following the trajectory of previous digital innovations that enhanced healthcare efficiency, chatbots have a promising future. Despite existing obstacles and some resistance, it's crucial to implement chatbots cautiously to ensure safety and avoid potential pitfalls.
